# Genetic mixing and demixing on expanding spherical frontiers

**DOI:** 10.1093/ismeco/ycae009

**Published:** 2024-01-22

**Authors:** Alba García Vázquez, Namiko Mitarai, Liselotte Jauffred

**Affiliations:** The Niels Bohr Institute, University of Copenhagen, Blegdamsvej 17, DK-2100 Copenhagen O, Denmark; The Niels Bohr Institute, University of Copenhagen, Blegdamsvej 17, DK-2100 Copenhagen O, Denmark; The Niels Bohr Institute, University of Copenhagen, Blegdamsvej 17, DK-2100 Copenhagen O, Denmark

**Keywords:** biofilm, morphology, E. coli, range expansion, spatio-genetic patterning, 3D growth

## Abstract

Genetic fluctuation during range expansion is a key process driving evolution. When a bacterial population is expanding on a 2D surface, random fluctuations in the growth of the pioneers at the front line cause a strong demixing of genotypes. Even when there is no selective advantage, sectors of low genetic diversity are formed. Experimental studies of range expansions in surface-attached colonies of fluorescently labelled micro-organisms have contributed significantly to our understanding of fundamental evolutionary dynamics. However, experimental studies on genetic fluctuations in 3D range expansions have been sparse, despite their importance for tumour or biofilm development. We encapsulated populations of two fluorescent *Escherichia coli* strains in inoculation droplets (volumes $\sim 0.1$ nl). The confined ensemble of cells grew when embedded in a hydrogel—with nutrients—and developed 3D colonies with well-defined, sector-like regions. Using confocal laser scanning microscopy, we imaged the development of 3D colonies and the emergence of sectors. We characterized how cell concentration in the inoculation droplet controls sectors, growth rate, and the transition from branched colonies to quasi-spherical colonies. We further analysed how sectors on the surface change over time. We complement these experimental results with a modified 3D Eden growth model. The model in 3D spherical growth predicts a phase, where sectors are merging, followed by a steady increase (constant rate), and the experimentally analysed sectors were consistent with this prediction. Therefore, our results demonstrate qualitative differences between radial (2D) and spherical (3D) range expansions and their importance in gene fixation processes.

## Introduction

In nature, bacteria and other single cellular organisms live in communities and often form dense structured populations such as colonies or biofilms. The structure, function, and stability of these communities depend on a complex network of social interactions, where bacteria exchange signals and metabolites, and protect each other from toxins, while at the same time proliferating and competing for space. This continuous cooperation and competition in the communities leads to complex spatial structures.

The structure has been widely investigated in surface-colonizing microbial populations (see [1] for a thorough review). These so-called competition experiments, in which well-mixed populations of bacteria (or yeast) [2] are inoculated on agar-surfaces and incubated, allow the cells to grow and divide and ultimately form complex macroscopic patterns [3]. When the initial founder cells are a mixture of differently coloured fluorescent cells, one can observe patterns of spatially segregated lineages, even among bacteria of similar (i.e. neutral) fitness. It is well known that the emergence of these sector-like regions is driven by random fluctuations at the outermost band of the expanding frontier [4], and also that the sector boundaries are diffusive [2, 5] and depend on, for example, environmental conditions [6, 7], extracellular matrix production [8], and cell shape [9]. Furthermore, the initial inoculating concentration has been found to control segregation patterns. In particular, the average sector regions’ sizes correlate with inoculation density [10–14], as space limits proliferation during range expansion [15]. These observations provide us with a deeper understanding of the population structure in biofilm and also serve as the foundation to understand the genetic drift and fixation in expanding populations in 2D [4, 16, 17].

Bacteria also live in 3D habitats, often in dense environments, where they are mired in mucus or entangled in polymers secreted by other bacteria, algae, or animal tissue. One model system of bacteria living in such settings is monoclonal spherical colonies in hydrogel [18]. The system has been used to investigate, for example, growth [19, 20], colony morphology [21, 22], quorum sensing [23], and phage sensitivity [24].

Despite these advances in 3D model systems and the broad interest in competition and evolution in microbial communities as well as tumour growth [17, 25], we still lack a suitable experimental model system to study competition in growing 3D bacterial colonies. Here, we propose such a system. We used a 1:1 co-culture of two fluorescently labelled *Escherichia coli* strains, which we encapsulated in agarose beads. We submerged these inoculation beads in a semi-solid agar (0.5%) and incubated them for a 3D competition assay. We imaged the resulting colonies using confocal laser scanning microscopy (CLSM), to know how the density of founder cells impacts growth and pattern formation. We further analysed how the number of sectors on the colony surface changes with time. Our findings are complemented with mathematical modelling to provide insight into spatial segregation in 3D communities. In particular, we demonstrate that the sector splitting, which we also observe in our experiment, becomes dominant in the long term. Our system serves as an effective tool to investigate the multi-species bacterial community and also as an ideal model system to analyse spatial population genetics in 3D growing colonies.

## Materials and methods

### Bacterial strains and culture media

We used two sub-populations of the *E. coli* B strain REL606 [26] carrying plasmids that constitutively expressed kanamycin resistance and either green fluorescent protein (GFP), pmaxGFP (pmaxCloning-Vector, Lonza), with excitation/emission 487/509 nm or red fluorescent protein (RFP), pTurboRFP (pmaxCloning-Vector, Lonza), with 553/574 nm [9]. Therefore, all media used in this study were supplemented with 30 $\mu $g ml$^{-1}$ kanamycin to retain fluorescence.

Through this study, we alternated between the following two media: the rich Lysogenic broth (LB) with 1% Bactotryptone, 0.5% NaCl, and 0.5% yeast extract and the M63 minimal media (M63+glu) with 20% 5$\times $M63 salt [27], 1 $\mu $g ml$^{-1}$ B1, 2 mM MgSO$_{4}$ and 2 mg ml$^{-1}$ (w/v) glucose.

### Encapsulating bacteria in inoculation beads

For a 3D competition experiment, we produced 2.5% agarose beads following the procedure of Buffi et al. [28]. One day prior to bead formation, pipette tips were placed in an incubator set to 60$^{\circ }$C, two overnight cultures of REL+GFP and REL+RFP in 2 ml of LB were prepared, and incubated at 37$^{\circ }$C under constant shaking. The following day, we measured (NanoPhotometer C40) the optical densities at the wavelength of 600 nm (OD) of the overnight cultures. Then, we prepared a saturated 1:1 mixture, by mixing the overnight cultures in the ratio corresponding to their ratio of the measured ODs. Meanwhile, 15 ml of silicone oil (dime-thylpolysiloxane, Sigma) in a 50 ml tube was heated in a block heater at 55$^{\circ }$C (AccuBlock Digital Dry Baths, Labnet International, Inc.) for $\sim 20$ min. Then, sterile 2.5% agarose (VWR Life Science, EC no:232-731-8, CAS No:9012-36-6) in Mili-Q water was melted in a microwave. We made sure to shake rigorously for homogeneity and to avoid boiling to minimize evaporation. Then, the agarose solution was left to cool to $\sim $55$^{\circ }$C, before 500 $\mu $l was transferred to a 2 ml (heated) tube in the block heater (55$^{\circ }$C). Afterwards, 15 $\mu $l of pluronic acid (Pluronic F-68 solution 10% Sigma) was added and the tube was transferred to a shaker block heater (Thermomixer Comfort, Eppendorf) set at 42$^{\circ }$C. After few minutes, 2–100 $\mu $l of the saturated 1:1 bacteria culture was added under continuous vortexing (1400 rpm) for final concentrations of OD $\in \{5.6,0.70,8.2\times 10^{-2},6.9\times 10^{-3}\}$ (Table 1). We removed the silicone oil from the block heater and—using the pre-heated pipette tips—500 $\mu $l of this bacteria–agarose–pluronic acid mix was added drop by drop to the silicone oil. This step was done fast to avoid untimely solidification of the agarose (at temperatures $<25^{\circ }$C). Afterwards, the 50 ml tube containing the droplets in silicone oil was vortexed (Vortex Mixer, Labnet International) for 2 min at maximum speed before being placed in a water-ice bath for additional 10 min. Then, centrifuged for 10 min at 550 g (room temperature), before the silicone oil was removed by careful pipetting followed by $2\times $ washing: (i) addition of 5 ml of phosphate buffered saline (PBS); (ii) centrifugation for 10 min at 550 g; and (iii) gentle removal of oil and supernatant. Finally, the washed beads were resuspended (by vortexing) in 5 ml of PBS.

**Table 1:** Inoculating bead’s concentration in units of OD or cells per ml, using the conversion OD$=1$ equal to $1.5\times 10^{9}$ cells per ml.

	OD	Cells per ml	Cells per bead
$c_{0}/100$	$6.9\times 10^{-3}$	$1.6\times 10^{7}$	1.0$\pm $0.7
$c_{0}/10$	$8.2\times 10^{-2}$	$1.21\times 10^{8}$	12$\pm $9
$c_{0}$	0.70	$1.06\times 10^{9}$	108$\pm $78
$c_{0}\times 10$	5.6	$8.46\times 10^{9}$	863$\pm $625

Notes: The fourth column is the calculated number of cells per bead, using the average volume, $({4}/{3})\pi \langle R_{\textrm{in}}\rangle ^{3}$ (Fig. 2B), and the error correspond to $\pm $SD propagated from the $\langle R_{\textrm{in}}\rangle $ error.

To harvest the beads, we let the bead–PBS solution flow through a set of strainers with mesh sizes of 70 $\mu $m (Cell strainer 70 $\mu $m Nylon, Corning) and 40 $\mu $m (Cell strainer 40 $\mu $m Nylon, Corning), placed on a 50 ml tube. An additional 5 ml of PBS was poured through the strainers to ease the passage. To collect the selected beads, the 40 $\mu $m strainer was placed upside down on a new 50 ml sterile tube and the beads adsorped to the filter surface were desorbed by rinsing with 6 ml of LB medium. For long-term storage, the bead-medium solution was aliquoted in volumes of 500 $\mu $l in 2 ml vials, before mixing with 500 $\mu $l of 50% glycerol-solution (11 vials per batch), and stored at −80$^{\circ }$C. We used 1 ml serological pipette tips for bead handling to prevent damaging flow rates.

### Growth rate measurements

Cultures of REL+GFP and REL+RFP were incubated (37$^{\circ }$C) in parallel overnight in 2 ml of M63+glu under constant shaking. The following day, 500 $\mu $l of each overnight culture were diluted in 5 ml of M63+glu and the ODs were measured over 5 hours with a sampling rate of $\sim 30$ minutes.

### Inoculation bead concentration measurements

#### Ratio of coloured strains

To verify the ratio in the beads (tuned with OD ratios), we serially diluted ($10^{6}\times $) the saturated 1:1 cell mixture (for encapsulation) and plated (50–200 $\mu $l) on LB plates with agar (1.5%). The plates were incubated overnight, and the number of CFUs from REL+RFP and REL+GFP was counted to verify the 1:1 ratio (Supplementary Figure S11).

#### Cells per bead

Overnight cultures of REL+GFP and REL+RFP were serially diluted ($10^{6}\times $) and plated (50 $\mu $l) on LB plates with 1.5% agar. From the number of CFUs, we found that OD $=1$ corresponds to $\sim 1.5\times 10^{9}$ cells ml$^{-1}$. Combined with OD$\in \{5.6,0.70,8.2\times 10^{-2},6.9\times 10^{-3}\}$ and the average bead volume, we estimated the average number of cells per bead given in Table 1.

#### Beads per volume

We mixed 80 $\mu $l of inoculation beads, either directly from production or from frozen vials, with 200 $\mu $l medium (LB). The mixture was plated by gently whirling (no spatula) on LB plates with 1.5% agar, then the plates were incubated overnight, and finally the number of CFUs was counted. This estimation of beads per volume was used to determine the proper dilution after thawing to ultimately get $\sim 20$ beads per well.

### Imaging inoculation beads

To estimate inoculation beads’ radii, beads were imaged with an inverted Nikon Eclipse Ti fluorescent microscope (Nikon, Tokyo, Japan) using a 20$\times $ air immersion objective (Splan flour,L20$\times $,0.45corr$\infty $) paired with an Andor Neo camera (Andor, Belfast, UK). GFP and RFP were excited by an Hg lamp using the FITC and Texas-red (Nikon, Tokyo, Japan) cubes, respectively.

### Toxicity measurements

To evaluate the toxicity of silicone oil and pluronic acid, we carried out limiting dilution experiments: overnight cultures of REL+GFP and REL+RFP were (10 - 10$^{7})\times $ diluted in 1 ml of either pluronic acid, silicone oil, or PBS as control. Then the number of viable cells was determined by plating (LB plates with 1.5% agar) followed by CFU counting.

### Competition assays

#### Embedment of inoculation beads in 0.5% agar

In line with methods detailed in [22] and references therein, bottles of 20 ml milipore water with 0.625% agar were melted by repeated cycles of heating (in the microwave) and shaking to ensure homogeneity and minimize evaporation and ageing [29]. After cooling ($\sim $55$^{\circ }$C), we added M63 salt [27] and supplements to a final M63+glu with 0.5% agar. Then, 1 ml of this solution was transferred to a pre-heated tube in the (non-shaking) block heater (55$^{\circ }$C). A frozen vial of bead-medium solution was thawed (on the bench) for $\sim $5 min until melted and 20–500 $\mu $l were transferred into 1 ml of LB (to minimize the time spent in high-concentration glycerol). Meanwhile, the agar-medium solution was supplemented with 10 $\mu $l of a 1 M stock of KNO$_{3}$—to avoid growth difficulties in anoxic conditions as suggested in [30, 31]—and 30 $\mu $g ml$^{-1}$ kanamycin. Quickly hereafter, 50 $\mu $l of the diluted bead-medium solution was added and the tube’s content was mixed and poured into a glass bottomed culture well (WillCo HBST-5040). Here it was left to solidify for a few minutes on the bench before incubation (upside down) at 37$^{\circ }$C for at least 5 hours (see imaging competition assays section). The agar had a final thickness of $\sim 300$$\mu $m and contained 17$\pm $5 inoculation beads per well (Supplementary Figure S12); corresponding to, at most, one bead for every $50$$\mu $l agar. For 3D competition experiments, the wells were incubated at 37$^{\circ }$C for 5–7 hours (depending on the experiment).

#### Deposition of inoculation droplets on 1.5% agar

In line with the protocol of Jauffred et al. [7] and references therein, we mixed 1:1 overnight cultures of REL+GFP and REL+RFP with LB to a final OD = 0.7 before inoculating 0.5 $\mu $l on plates with M63+glu and 1.5% agar. Plates were incubated for 20 hours at 37$^{\circ }$C.

### Imaging competition assays

#### 3D colony images

After incubation, we checked all culture wells to discard those where colonies had outgrown the agar and spread over the agar–air interface. All colonies of the remaining culture wells were imaged with CLSM (Leica SP5) and a 20$\times $ air objective (NplanL20$\times $0.40NA). The data were acquired with sequential z-stacks of RFP (488 nm laser, detection range of 498–536 nm) followed by GFP (543 laser, detection range of 568–641 nm) to a final image of voxel size in ($x,y,z$) of (1.51,1.51,1.33) $\mu $m. For every new condition, we made sure to collect images from at least two to three different wells.

#### Time-lapse 3D images

After 4–7 hours of incubation, our colonies had outgrown the inoculation beads and reached a diameter of $\sim 100$$\mu $m. For CLSM time lapses, our microscope was equipped with a standard water-based heating stage set to 37$^{\circ }$C and we carried out 5 hours of imaging with a frame rate of four per hour. We repeated this two to five times for each condition. We also investigated time-evolvement by comparing individual sets of colonies incubated for different amounts of time. The visualization was made using the Fiji 3D viewer plugin [32].

#### 2D colony images

The agar plate was placed upside down on our inverted confocal laser scanning microscope. They were imaged with sequential z-stacks using a similar procedure as for the 3D colonies but with an 5$\times $ air objective (Nplan5$\times $0.12PHO, Leica). The voxel size in ($x,y,z$) was (6.07,6.07,10.33) $\mu $m and we used the Pairwise Stitching [33] plugin for Fiji.

### Image analysis

#### Segmentation of 3D colonies

The 3D image segmentation was done using both BiofilmQ [34], Matlab and Fiji/Image J [35] and the following work flow. First, the segmentation was done separately for each channel (GFP+RFP) in BiofilmQ. We cropped the image stacks in the $z$-direction (same $z$ for both channels) to discard the colony half-sphere furthest away from the objective, which was distorted by strong shadowing effects. Then, we de-noised the images by convolution ([kernel size: [$x,y,z$] = [$5,5,3$] pixels) to soften the edges and fill the holes between cells. Using the Otsu method (with two classes: object and background), we manually selected the threshold value for each image in the stacks. Afterwards, using the Overlay function, we checked that the segmentation matched the raw data. Hereafter, the resulting masks of the channels (GFP+RFP) were divided into small cubic volumes of length 1.52 $\mu $m, which reflects the voxel size of the image and corresponds to 2–4 *E. coli* volumes. Hereafter, the channel masks were merged, and the surface of the resulting mask was found calling Cube_Surface followed by the Filter Objects function with Cube_Surface in the range [1, 2]. The resulting segmentation of the merged channels were saved (mat-file) and the Cube_Surface voxel indices vector was converted to a matrix (using Cell2mat). The matrix can be read as a 3D logical image of a connected surface, but what we wanted was a hollow half-sphere with the thickness of a single voxel. Therefore, we manually removed the surface furthest away from the objective using the Paintbrush tool in Fiji.

The resulting segmentation mask was multiplied separately with each segmented channels (GFP and RFP) and then merged to the resulting surface mask. We found the number of voxels in the surface mask that was assigned both colours (GFP and RFP) to be $(3\pm 2)$%, $(4\pm 1)$%, and $(11\pm 4)$% for $c_{0}/10$, $c_{0}$, and $c_{0}\times 10$, respectively (Supplementary Figure S9). As they are few and because RFP in our system is less detectable, we decided to assign all these two-coloured voxels to RFP. We also found that the surface mask had some discontinuities (<‰), using 3D Fill Holes plugin in Fiji [36]. Hence, the resulting surface mask, $M$, is a single layer of voxels each with one and only one colour (see an example in the Supplementary Movie 1).

#### Size estimation of 3D colonies

From $z$-projections of the mask $M$ (or the thresholded image of beads), we obtained logical ($x,y$)-images. The projected areas of the $n$th colony (bead), $A_{\textrm{prj}}^{(n)}$, was found by counting pixels $\neq 0$ and multiplying by pixel area conversion in ($x,y$). By assuming the colony (bead) to be spherical, we estimated the ensemble-averaged radius, $R$, at time $t$ to be


(1) \begin{align*}& \langle R(t)\rangle=\frac{1}{N\sqrt{\pi}}\sum_{n=1}^{N}\sqrt{A_{\textrm{prj}}^{(n)}(t)},\end{align*}

where $N$ is the total number of colonies.

#### Determining perimeter in 3D bacterial colonies

From $z$-projections of the mask $M$ (or the manual thresholded images of the $z$-projections), the projected perimeter, $P$, of the $n$th colony was found by using the Fiji function called Analyze Particles.

#### Isoperimetric quotient

Measuring the perimeter, $P$, of $A_{\textrm{prj}}(t)$ for a projection of a colony, we found the ratio between $A_{\textrm{prj}}(t)$ and the area of a circle with the same $P$, called the isoperimetric quotient for a colony to be


(2) \begin{align*}& \rho_{\textrm{prj}}(t) = 4\pi\frac{A_{\textrm{prj}}(t)}{P^{2}},\end{align*}

which is a dimensionless measure of compactness with the maximum compactness for a perfect circle ($\rho =1$). However, because of square pixels that roughen the shape boundaries, we normalized each value of $\rho _{\textrm{prj}}(t)$ with the $\rho (t)^{\prime}$ corresponding to a pixel-resolved circle with an area $\sim A_{\textrm{prj}}(t)$. In detail, we found $\rho (t)^{\prime}$ of a circle with pixelated boundaries circle with the area: $A^{\prime}=A_{\textrm{prj}}$ and perimeter $P^{\prime}$. The result is then


(3) \begin{align*}& \rho(t)=\frac{\rho_{\textrm{prj}}(t)}{\rho^{\prime}(t)}.\end{align*}

#### Determining occupancy in 3D bacterial colonies

We defined the ensemble-averaged occupancy of GFP-expressing bacteria, $\mathcal{O}$, on the colony surface as the sum over the following ratio:


(4) \begin{align*}& \langle\mathcal{O}(t)\rangle=\frac{1}{N}\sum_{n=1}^{N}\frac{A_{\textrm{GFP}}^{(n)}(t)}{A^{(n)}(t)}.\end{align*}

Here, $A_{\mathrm{GFP}}^{(n)}(t)$ is the area of GFP-expressing bacteria and $A^{(n)}(t)$ is the total surface area of the $n$th colony at time $t$, such that $A^{(n)}(t)=A_{\mathrm{GFP}}^{(n)}(t)+A_{\mathrm{RFP}}^{(n)}(t)$.

#### Sectors in 3D bacterial colonies

We defined 3D sectors as connected regions on the colony surface using the Fiji plugin Find Connected Regions on the colony mask $M$ with a minimum sector area of 10 voxels ($\sim $23 $\mu $m$^{2}$). This value was chosen to be optimal for our dataset by comparing the automated sector counts, $\sigma (t)$, with manual counts. The area of a sector (GFP or RFP) at a given $t$ is $A_{\mathrm{sec}}(t)$, which we found by scaling the number of voxels in the sector with the conversion in ($x,y$). We plotted them as so-called survival curves, which is the cumulative probability of finding $A_{\mathrm{sec}}(t)$ (for a specific $t$) larger than a specific value of the area, $a$: $\sum _{a_{i}>a}A_{\mathrm{sec}}^{a_{i}}(t)/\sum _{i}A_{\mathrm{sec}}^{(i)}(t)$, where $i$ is the integer of all sectors in all colonies.

We also found the ensemble-averaged number of connected sectors (GFP+RFP) on the colony surface,


(5) \begin{align*}& \langle\sigma(t)\rangle=\frac{1}{N}\sum_{n=1}^{N}\sigma^{(n)}(t),\end{align*}

where $\sigma ^{(n)}(t)$ is the number of sectors on the colony mask of the $n$th colony at a given $t$.

For the modelled colonies, we used the minimum sector area of one lattice site, as there is no ambiguity in the assignment of colours in this case.

### 3D Eden growth model

We employed 3D Eden growth model to simulate bacterial colonies growing from an inoculation bead in a 3D environment. The model divides the space into a cubic lattice, where each lattice site can be occupied by “bacteria” or empty, and the growth happens if there is an empty neighbour site. It should be noted that the correspondence of the length- and time-scale between the model and the experiment is only qualitative; this is a coarse-grained model where one occupied lattice site represents a meta-population of a cluster of several cells [17].

#### Initial condition two-species population

In a 3D cubic-lattice of size $(L\times L \times L)$ with $L=200$, we placed (in the centre) a sphere of radius, $R_{\mathrm{s}}=19$ lattice sites. Individual seeds were placed randomly inside the sphere and the number of seeds were on average $N \in \{16,144,1150\}$ drawn from a 1:1 distribution of the two colours of seeds (one or two), corresponding to seeding concentrations of $m/10$, $m$, and $m\times 10$. More specifically, we filled each site inside the sphere with the probability $N/V$ with $V=({4}/{3})\pi R_{\mathrm{s}}^{3}$, with one of the two colours assigned with equal probability.

#### Initial condition multi-species population

The initial conditions were the same as for the two population model, except that each seed was different: $[1,\ldots ,\langle N\rangle ]$ with equal probability ($N/V$).

#### Update rule

We first make a list $S$ containing all surface sites, that is, occupied sites with one or more empty neighbouring sites out of the six neighbours. The growth is done as follows.

(i) Choose one site to divide from the sites in the list $S$ randomly with equal probability.
(ii) Randomly choose one of the empty neighbour sites of the chosen site in (i).
(iii) Fill the chosen site in (ii) with the same colour as the chosen site in (i).
(iv) Update the list $S$ such that (a) the new site was added to $S$ if it had one or more empty neighbouring sites and (b) the sites were removed from $S$ if they no longer had any empty neighbouring sites.

We repeated (i)–(iv) $5\times 10^{5}$ times. For every division, the time proceeds by the inverse of the number of surface sites in $S$, meaning that a unit of time corresponds to the “generation time” (an occupied site on the surface duplicates on average once per unit of time).

We coded the model in C++ using a C++ library Armadillo ([37]).

### Statistics

All mean values are given as mean plus/minus the standard error of the mean (SEM) or the standard deviation (SD) and only when data are tested against the null hypothesis that it is normally distributed. For distributions, bin sizes were chosen following Sturge’s rule, unless stated otherwise.

## Results

**Figure 1:**
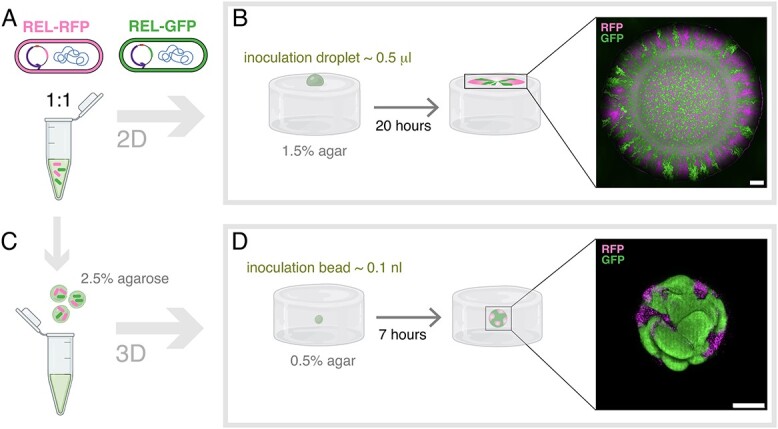
Competition experiments in 2D and 3D. Notes: (A) The 1:1 mixture of fluorescent *E.coli* REL coding for either GFP or RFP with concentration $c_{0}$ or multitudes of $c_{0}$. (B) Schematic diagram of competition experiment in 2D: inoculation of a 0.5 $\mu $l inoculation droplet on M63+glu agar (1.5%) plate, followed by 20 hours of incubation (37$^{\circ }$C). The inset is a (maximum intensity) z-projection of an example colony imaged by CLSM. The scale bar corresponds to 50 $\mu $m. (C) Inoculation beads: small agarose (2.5%) beads encompassing the 1:1 cell mixture from (A). (D) Schematic diagram of competition experiment in 3D: inoculation of a 0.1 nl inoculation bead inside a M63+glu agar (0.5%), followed by 7 hours of incubation (37$^{\circ }$C). The inset is a (maximum intensity) z-projection of an example colony imaged by CLSM. The scale bar corresponds to 50 $\mu $m. Elements of this figure were created with BioRender.com.

We mixed two sub-populations of the non-motile *E. coli* B strain (REL) carrying plasmids coding for either green fluorescent protein (GFP) or red fluorescent protein (RFP) in 1:1 ratio, as shown in Fig. 1A. We verified that the cost of fluorescence-coding plasmid (i.e. growth rate) was similar for the two sub-strains. The generation times in the exponential growth phase in an M63 minimal medium supplemented with glucose (M63+glu) were (59$\pm $1) min and (60$\pm $1) min for the GFP and RFP carrying sub-strains, respectively (Supplementary Figure S1).

When a mixture of bacteria is inoculated on an agar surface, the population expands and segregates into monoclonal sectors. Fig. 1B is a sketch of such a (pseudo-)2D competition assay, where 0.5 $\mu $l droplets of the 1:1 mix were inoculated on agar (1.5%) supplemented with nutrients. In line with prior findings [4], the expansion led to strong demixing of the two strains (inset of Fig. 1B). In order to set up a 3D version of this experiment, we designed multi-cell beads based on a protocol by Roelof van der Meer and co-workers [28]. This method, which is sketched in Fig. 1C, relies on the hydrophobic nature of silicone oil to form small droplets of an aqueous solution. In our case, this solution is a mixture of bacteria and melted agarose (2.5%). We used this strategy to encapsulate the *E. coli* mixture (Fig. 1A) in inoculation beads. When embedded in a semi-soft (0.5%) agar supplemented with nutrients and incubated (37$^{\circ }$C), colonies outgrow the original agarose sphere to form 3D bacterial colonies as sketched in Fig. 1D. The inset is an example of a colony derived from an inoculation bead with 1:1 mixture of fluorescent bacteria of concentration $c_{0}$, which corresponds to $\sim $100 bacteria at the onset of the colony (Table 1).

### Inoculation bead characterization

**Figure 2:**
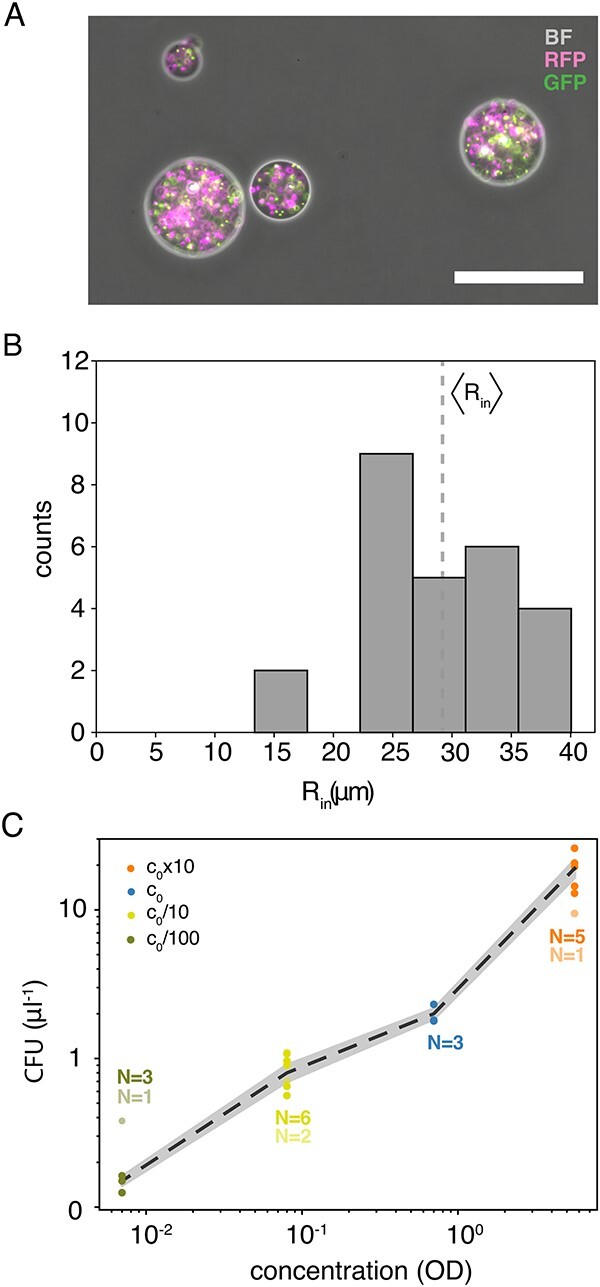
Inoculation bead characterization. Notes: (A) Inoculation beads with $c_{0}$ concentration (Table 1). The image is an overlay of channels: bright-field (BF), green (GFP), and red (RFP). The scale bar is 100 $\mu $m. (B) Distribution of inoculation beads’ radii, $R_{\mathrm{in}}$, with the average value (vertical dotted line): $\langle R_{\mathrm{in}}\rangle =(29\pm 7)$$\mu $m (mean$\pm $SD, $N=26$) as given in equation 1. (C) CFUs corresponding to the number of inoculation beads per volume versus density of founder cells (i.e. OD: $c_{0}/100$, $c_{0}/10$, $c_{0}$, and $c_{0}\times 10$ on a double-logarithmic scale). Beads are either from frozen stocks (full colour) or fresh from production (opaque); the latter are not included in the mean (punctuated line) and the shaded area corresponds to $\pm $SEM.

Using wide-field fluorescence microscopy, we found that the spherical inoculation beads contain bacteria of both colours, as seen in Fig. 2A. We found no bacteria outside the beads, implying that the agarose scaffold protects the cells from the toxic silicone oil (Supplementary Figure S2). Furthermore, we found that their radii, $R_{\mathrm{in}}$, were distributed as given in Fig. 2B, with average $\langle R_{\mathrm{in}}\rangle =(29\pm 7)$$\mu $m (mean$\pm $SD, $N=26$), which corresponds to a volume of about $0.1$ nl. Hence, the inoculation volume is on the order of $10^{3}$ times less than in the 2D case (0.5 $\mu $l). Our different batches of inoculation beads contained a 1:1 mix of the two colours (GFP and RFP) of bacteria and varying concentrations of cells: $c_{0}\times 10$, $c_{0}$, $c_{0}/10$, and $c_{0}/100$ (Table 1). In order to estimate the concentration of beads that are able to grow into colonies, we plated the beads on a hard agar surface, incubated the plates, and counted the number of colony-forming units (CFUs). Fig. 2C combines the counts fresh from production (opaque) and from frozen stocks (full colour). We found significant differences in CFU per volume for beads of different initial cell concentration. However, in practice, we tuned the bead solution to have approximately the same amount of growing colonies per culture well.

### Density of founder cells controls size and patterning

**Figure 3:**
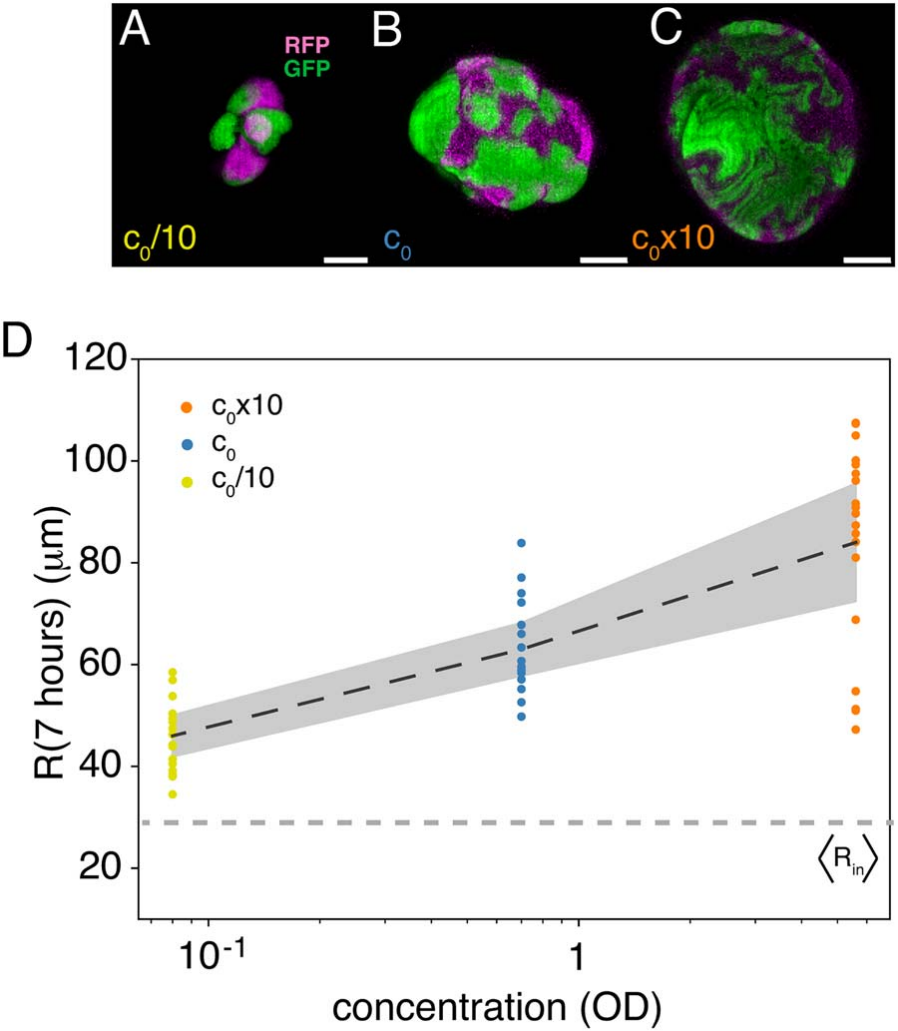
Density of founder cells controls size and patterning. Notes: (A–C) Examples of colonies formed from inoculation beads with different initial concentrations of founder cells: (A) $c_{0}/10$ (A), (B) $c_{0}$, and (C) $c_{0}\times 10$. The scale bars correspond to 50 $\mu $m. (D) Colony radii, $R(t)$, at $t=7$ hours for $c_{0}/10$ ($N=18$), $c_{0}$ ($N=17$), and $c_{0}\times 10$ ($N=19$). The dashed line (dark grey) is the ensemble average (equation 1) and the shaded region corresponds to $\pm $SEM. The horizontal dashed line (light grey) is the average bead size, $\langle R_{\mathrm{in}}\rangle $ (Fig. 2B).

**Figure 4:**
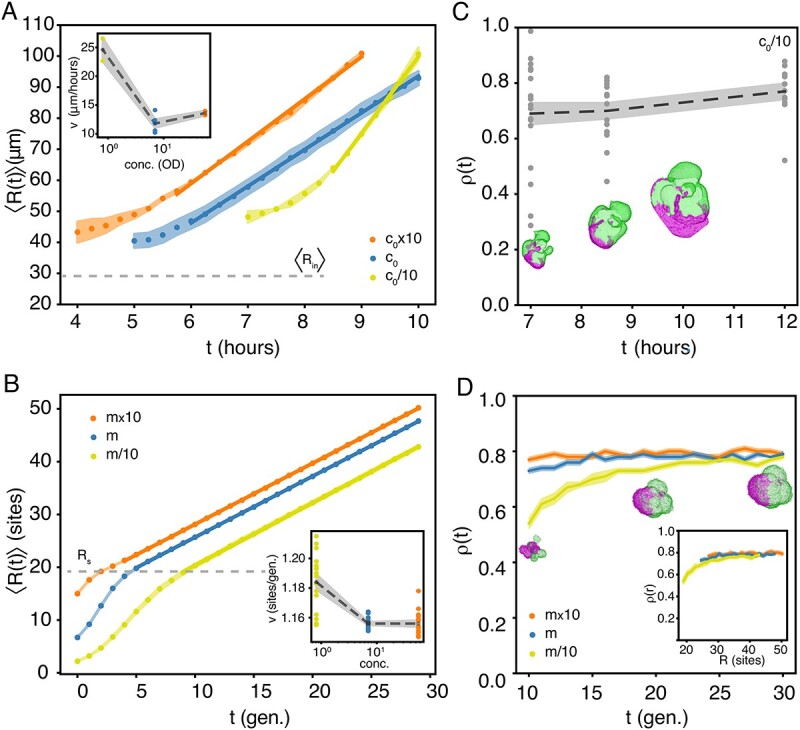
Density of founder cells controls growth dynamics. Notes: (A) Ensemble-averaged colony radii, $\langle R(t)\rangle $, versus time (equation 1) for $c_{0}/10$ ($N=2$), $c_{0}$ ($N=5$), and $c_{0}\times 10$ ($N=2$). The full line is a linear fit and the shaded areas signify $\pm $SEM. The dashed line is the mean radius of the inoculation beads, $\langle R_{\mathrm{in}}\rangle $, found from Fig. 2B. The inset shows radial growth speeds, $v$, of the individual traces versus concentration. The dots are the linear fits of the individual $R(t)$ from the colonies in (A), the dashed line is the mean, and the shaded area signifies $\pm $SEM. The dots are the slopes of the individual time lapses. (B) Ensemble-averaged *in silico* colony radii, $\langle R(t)\rangle $, versus time (equation 1) for $m/10$ ($N=15$), $m$ ($N=15$), and $m\times 10$ ($N=15$). The full line is a linear fit and the shaded area signifies $\pm $SEM. The dashed line is the mean radius, $R_{\mathrm{s}}$, of the initial seeding sphere (i.e. inoculation beads). The inset shows radial growth speeds, $v$, of the individual traces versus concentration. The dashed line is the linear fit of the full line and the shaded area signifies $\pm $SEM. The dots are the slopes of the individual time lapses. (C) Isoperimetric quotient, $\rho (t)$ (equation 3), for different $c_{0}/10$ colonies at different time points, $t$: 7 hours ($N=18$), 8.5 hours ($N=16$), and 12 hours ($N=12$). The dashed line is the mean and the shaded area signifies $\pm $SEM. The insets are the masks, $M$, from a time trace from (A). (D) Isoperimetric quotient, $\rho (t)$, for growing *in silico* colonies ($N=15$) of concentrations $m/10$, $m$, and $m\times 10$ over time, $t$. The full line is the mean and the shaded area signifies $\pm $SEM. The small insets are examples from one model evaluation with concentration $m/10$. The inset shows the isoperimetric quotient, $\rho (r)$, over radial distance, $R$.

**Figure 5:**
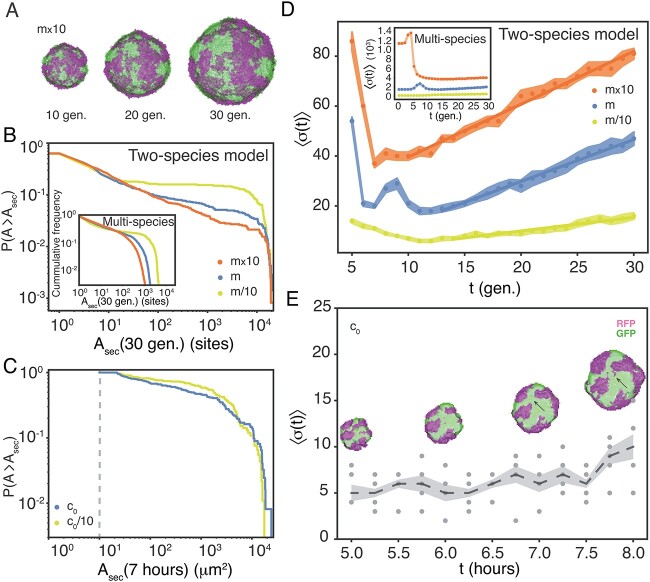
Sector patterns are dynamic. Notes: (A) An example of how surface sectors change with time, $t\in \{10,20,30\}$ generations in a two-species $m\times 10$-derived modelled colony. (B) Cumulative distributions of areas of sectors, $P(A\ge A_{\mathrm{sec}})$, of sub-population on the two-species *in silico* colonies’ surfaces, $A_{\mathrm{sec}}(t)$, at $t=30$ generations for $m/10$ ($N=233$), $m$ ($N=702$), $m\times 10$ ($N=1219$) sector from 15 different colonies. The inset shows the multi-species model for $t=30$ generations for $m/10$ ($N=708$), $m$ ($N=2978$), and $m\times 10$ ($N=5939$) sectors from 15 different colonies. (C) Cumulative distributions of areas of sectors, $P(A\ge A_{\mathrm{sec}})$, of sub-population on the experimental colonies’ surfaces, $A_{\mathrm{sec}}(t)$, at $t=7$ hours for $c_{0}/10$ ($N=92$) sectors, as well as $c_{0}$ ($N=123$) sectors from 18 and 17 colonies, respectively. The vertical dashed line signifies the detection limit of 10 voxels. (D) Ensemble-averaged number of sectors, $\langle \sigma (t)\rangle $, versus time, $t$ (equation 5) for concentration: $m/10$ (yellow), $m$ (blue), and $m\times 10$ (orange) as given in equation 5. There is no distinction between the two colours of sectors and the number of colonies is ($N=15$). The splitting rate found by linear fit of the linear part is $0.55\pm 0.04$ gen$^{-1}$, $1.68\pm 0.04$ gen$^{-1}$, and $2.12\pm 0.06$ gen$^{-1}$ for $m/10$, $m$, and $m\times 10$, respectively. Meanwhile, the density of sectors reaches a constant low level (Supplementary Figure S8A). The shaded area signifies $\pm $SEM. The inset shows the same for the multi-species model, where the splitting rate is $1.03\pm 0.07$ gen$^{-1}$, $3.56\pm 0.13$ gen$^{-1}$, and $2.35\pm 0.17$ gen$^{-1}$ for $m/10$, $m$, and $m\times 10$, respectively. Also for this model, the density of sectors reaches a constant low level (Supplementary Figure S8B). (E) Ensemble-averaged number of sectors, $\langle \sigma (t)\rangle $, versus time, $t$ (dashed line) as given in equation 5 for $c_{0}$ ($N=5$). The dots are the number of sectors for individual colonies without distinguishing between red and green. The shaded area signifies $\pm $SEM. The inset shows the time evolution of surface sectors for a $c_{0}$-derived example colony. An example of sector splitting is designated (black arrows).

Following agar-embedment and incubation, we imaged colonies using CLSM. We noted how often we found monocolour colonies (Supplementary Figure S3) but only imaged two-coloured colonies. Fig. 3A–C shows three examples of colonies grown from different batches of inoculating beads—(A) $c_{0}/10$, (B) $c_{0}$, and (C) $c_{0}\times 10$—and incubated for 7 hours (37$^{\circ }$C). At first sight, we found that not only does the size of the colony change with concentration, but also the patterning (more examples in Supplementary Figure S4). For a thorough analysis of this general observation, we segmented and filtered the 3D images to obtain a mask—with the thickness of a single voxel—reconstituting a connected surface and where all voxels were assigned one (and only one) colour (see the insets of Figs 4 and 5 and Supplementary Movie 1 for examples). From this mask, we estimated the time-dependent radius, $R(t)$ for $t=7$ hours, of the projected surface (assuming spherical symmetry). In line with the general observation, $R(t)$ grows with the density of founder cells, as shown in Fig. 3D.

### Density of founder cells controls growth dynamics

To study the dynamics of the 3D bacterial colony formation, we did time lapses (Supplementary Movie 2) and Fig. 4A shows the average radii, $\langle R(t)\rangle $, for the three concentrations: $c_{0}/10$ (yellow), $c_{0}$ (blue), and $c_{0}\times 10$ (orange), as defined by equation 1. The time point at which we began imaging was chosen such that we easily found the small colonies under the microscope ($R>40$$\mu $m) and we ended at $\sim 10$ hours to prevent outgrowing the matrix. Generally, we find an initial growth phase, where the colony radius expands slower than linear over time. This is followed by a temporal window, where $\langle R\rangle $ grows linearly over time (full lines) with the rate, $v$, given in the inset of Fig. 4A. This linear expansion indicates that the colonies grow predominantly from the outermost cell layers due to insufficient nutrient penetration deeper in the colony [38]. Also, this is well in accordance with earlier predictions [39, 40] and experimental findings for a similar *E. coli* system [22]. Notably, from these earlier predictions, we would expect $v$ to be similar for all concentrations, as is the case for $c_{0}$ and $c_{0}\times 10$. However, our results from the lowest density of founder cells ($c_{0}/10$) contradict this view (see the inset of Fig. 4A). Instead, the radial range expansion rate is significantly faster ($v\sim 25$ vs. $\sim 13$$\mu $m per hour). Assuming the doubling time to be $\sim 1$ hour (also inside 0.5% agar) and the volume of a single cell to be $\sim 1$$\mu $m$^{3}$, this corresponds to a growing layer of the thickness of about 25 cells.

As it is unclear how fewer inoculating cells result in faster radial growth, we explored this question employing an Eden growth lattice model initiated from two species of seeds/cells with identical properties randomly placed in a sphere of radius, $R_{\mathrm{s}}$, of three different concentrations: $m/10$, $m$, and $m\times 10$ (see the “Materials and methods” section). These seeds were allowed to divide into empty unoccupied neighbouring sites until they eventually grew as one cluster [41]. The resulting pattern mimicked the competition of two populations in confined bacterial colonies. To study the dynamics of the resulting *in silico* colony formation (Supplementary Figure S5), we created time-lapse movies of growing colonies with different seeding concentrations (Supplementary Movies 3–5). Fig. 4B shows the average radii, $\langle R(t)\rangle $, for the three concentrations: $m/10$ (yellow), $m$ (blue), and $m\times 10$ (orange) in the linear growth part of the curve as defined by equation 1. In contrast to the experimental observation, we observed the initial growth ($\langle R(t)\rangle <R_{\mathrm{s}}$) in the simulation to be faster and settling to linear growth with a constant rate (see the inset of Fig. 4B). We find large variations in $v$, especially for the lowest seeding concentration ($m/10$). Moreover, the radial velocity is largest for this concentration (even though the difference is small) because of rougher surfaces.

In the Eden growth model, the expansion speed is dominated by the number of cells on the surface (i.e. cells neighbouring empty lattice sites). Because colony shapes are rougher for lower concentrations of founder cells and for earlier times (Fig. 4D), the radial growth at earlier times will be faster. This effect is especially visible when the colony is smaller than the seeding sphere ($\langle R(t) \rangle \le R_{\mathrm{s}}$) and the occupied lattice sites are not yet fully connected. Moreover, the colony’s morphology could also cause fast growth of the colonies initiated from $c_{0}/10$ inoculation beads in the experiment. In particular, protrusions on the surface enlarge the surface-to-volume ratio and may enhance growth by making nutrients accessible to a larger number of cells. To investigate this, we measured the time-dependent isoperimetric quotient, $\rho (t)$ (equation 3), for the projection of $c_{0}/10$ colonies, as shown in Fig. 4C (see the “Materials and methods” section). $\rho (t)$ is a dimensionless measure of compactness and approaches its maximum for a perfect circle projection. We found large scattering in $\rho (t)$, especially for small $t$, but the average $\rho (t)$ tends to grow slightly, as the cracks and valleys are filled and colonies become more round. For comparison, in Fig. 4D, we measured $\rho (t)$ for the modelled data for $t\geq 10$ generations, when the colony is one connected structure and $\langle R(t)\rangle $ shows linear behaviour over time (Fig. 4B). For the modelled data, we found that $\rho (R)$ is independent of inoculation density (see the inset of Fig. 4D) and that it converges to a value comparable to the experimentally observed $\rho (t)$.

### Occupancy on colony surfaces matches the initial ratio in the inoculation bead

In order to evaluate any competitive advantage for one sub-population over the other, we measured the occupancy, $\mathcal{O}$(7 hours), or the fraction of the GFP-expressing sub-strain on the colony surface (equation 4). We found equal occupancy of two sub-populations, as the fraction of the surface occupied by GFP-carrying cells is centred around $\mathcal{O}=1/2$ for all inoculation bead concentrations (Supplementary Figure S6). As the sub-populations have equal fitness advantage (Supplementary Figure S1), the final surface ratio corresponds to the 1:1 mixture in the inoculation bead. Also, we find from our model that time-dependent fluctuations in $\mathcal{O}(t)$ are more important for smaller inoculation concentrations (Supplementary Figure S7).

### Sector patterns are dynamic

With the aim of characterizing the sectors on the colony surfaces and their time dependence, we identified the different sectors on the 3D *in silico* colony surfaces, as green and magenta in the examples in Fig. 5A. Then we calculated their time-dependent areas, $A_{\mathrm{sec}}(t)$, on the surface (see the “Materials and methods” section). From the cumulative frequency, as shown Fig. 5B, at a specific time point ($t=30$ generations), we found sectors to be larger for low seeding density. With this version of the Eden growth model, we lose track of the individual lineages. Therefore, we ran a modified model in parallel (Supplementary Figure S8 and Supplementary Movies 6–8), which grew from multi-seeds (see the “Materials and methods” section). From the cumulative frequencies (see the inset of Fig. 5B) we found that the majority of sectors are smaller than 10 sites and that the largest sectors indeed are merges of sectors of different lineages. We complemented this finding with the $A_{\mathrm{sec}}(t)$ at $t=7$ hours for both $c_{0}/10$- and $c_{0}$-derived colonies, as shown in Fig. 5C. Even though our detection limit was 10 voxels ($\sim 370$$\mu $m$^{2}$), we found that smaller sectors dominated. We left $c_{0}\times 10$ colonies out of the analysis, as many sectors were smaller than our detection limit and resolving colours was difficult (Supplementary Figure S9).

We set out to investigate the time dependence of the number of sectors, $\sigma (t)$, using our Eden growth models. As shown in Fig. 5D, the average $\langle \sigma (t)\rangle $ first decreases, as a consequence of merging of clusters from different founder cells (note that we counted spatially separated clusters as different sectors), followed by a linear increase in sectors, due to sector splitting. The merging (i.e. $\langle \sigma (t)\rangle $ decrease) is even more pronounced for the multi-species model (see the inset of Fig. 5D), even though subsequent splitting rates are larger.

We finally looked into how the number of sectors changes with time, $\sigma (t)$, in $c_{0}$-derived colonies in Fig. 5C. Even though we expected more splitting events for $c_{0}\times 10$, again, most sectors were below our resolution. Based on the time lapses of five colonies, we found that the ensemble-average, $\langle \sigma (t)\rangle $, as defined in equation 5, goes slightly up within our time range. This corresponds to a splitting of sectors, as shown for one example in Fig. 5E (black arrow). In contrast, the sector merging was not possible to observe experimentally because it happened inside the beads right after embedment ($t<5$ hours) and we were limited by our optical resolution. Once the clusters are connected, the cells grow as one colony and hereafter sector splitting dominates, giving an increase in $\langle \sigma (t)\rangle $. It is worth noting that there is no obvious way to calibrate the experimental time-scale against the simulation time-scale, as we ignore the spatial interpretation of the model’s lattice sites.

## Discussion

Recent advances in 3D culture technology allow for *in vitro* models of development and disease with organoids (i.e. simplified organs) [42] or tumourospheres [43, 44], respectively. The counterpart of these 3D models within bacteriology research is quasi-spherical colonies, grown from a few bacteria embedded in agar. We propose 3D cultures initiated from agarose droplets could be this simple (yet highly controllable) multi-species model of biofilm-formation in soil, natural water environments, minerals or other substrates.

In this paper, we have presented a method that, in brief, substitutes the inoculation droplet with an inoculation bead of much smaller volume (fractions of $\mu $l versus nl). So, our colonies are initiated from very small volumes and thus we can inquire about the early stages of colony development. Furthermore, we interchanged the hard agar ($>1\%$) substrate with submersion in soft agar ($<1\%$), which allows for studies of spherical range expansion. We used CLSM for imaging, but other techniques to optimize the resolution and light penetration in large cell agglomerates are evolving rapidly (e.g. light-sheet microscopy [45]). Even though advanced imaging is required for proper 3D reconstitution, we anticipate that many questions could be answered by 3D competition assays under wide-field fluorescence microscopy.

From 2D competition experiments, we know that sector boundaries are diffusive [4] and for rod-shaped cells even super-diffusive [2]. The reason for this is that uni-axial growth of rod-shaped bacteria (i.e. *E. coli*) results in chain formation of bacteria. As compressing forces make small asymmetries in cell alignment, these instabilities propagate to cause jagged shapes on surface-attached monolayers [46, 47]. These shapes are further enhanced by inter-cellular adhesion, which leads to increased diffusivity of mixing and area of interaction between lineages [48]. We find similar patterns of jagged sector boundaries on the colonies’ surfaces (Fig. 3C), even though our boundaries are 2D interfaces between sectors. This highlights an interesting follow-up question: how does cell alignment affect the spatio-temporal dynamics of sectors?

The Eden growth model predicts a transition from super-linear growth in early colonies to slower (i.e. linear) surface expansion over time (Fig. 4B). The super-linear regime largely overlaps with the regime where the growth—starting from the individual founder cells—is not yet connected into one colony. Our experimental resolution did not allow us to observe such early super-linear dynamics and, instead, we observed a slower growth. We speculate that this slow growth could be due to a possible heat shock from taking the culture wells out from the incubator to the CLSM for imaging the colonies and/or due to the higher mechanical stress the cells might be under when growing inside the agar beads (2.5% agarose) and that this stress reduces once cells have outgrown the beads and expand in the agar matrix, allowing them to growth faster. We did observe the linear surface expansion in experiments. The reason for this linear regime is that behind the fast-growing surface cells is a quiescent region, where proliferation is slower because of space constraints [49] and metabolite diffusion from the colony periphery [39, 40, 50]. We also find that the entrance into the linear regime depends on the initial concentration of founder cells, where the colonies with the highest initial concentration reach this compact-mature colony state earlier (Fig. 4A). This is also consistent with the simulations (Fig. 4B).

We also found that, for the lowest density of founder cells, the colonies have a rougher surface growth as earlier reported [51]. The cells located in these bumps or protuberances are expected to have better availability of nutrients, which can be part of the explanation for their observed faster radial growth (see the inset of Fig. 4A). The model also showed slightly faster growth for lower initial cell density (see the inset of Fig. 4B), though the difference was small. Furthermore, we believe that the surface roughness decreases slowly with time and the colonies become rounder and rounder (Fig. 4C). For 2D colony growth, it has been established that colony morphology qualitatively changes with nutrient availability and agar hardness. Specifically, a nutrient-poor environment and/or harder agar can cause instabilities to develop branching [52]. Similar instabilities are expected in 3D growth under certain environmental conditions. The observed lack of strong instabilities in our system supports the use of the Eden growth model that ignores inhomogeneity and nutrient depletion of the environment. Our model presumes homogeneity inside the colony. We speculate that the merging of somewhat grown micro-colonies may lead to structural variations and affect the overall growth. Hence, to understand the growth dynamics quantitatively, a model that considers these factors would be desirable.

We have demonstrated that the sectors on the surface can merge but also split over time. In our experiments, we observed the splitting events, and the number of sectors showed a tendency to increase, though fluctuation was large (Fig. 5C). These observations are consistent with the multi-species Eden growth model simulation and independent of motility of individual cells (as our strain lacks flagella [53]). In 3D expanding colonies, individual clusters—starting from individual seeds—quickly merge to form a connected colony, and after that, although large sectors do form, they also fragment, forming small sectors. Therefore, the number of sectors actually increases over time (Fig. 5E). This contrasts with what we know from 2D radial expansions, where an initial coarsening phase, in which domain boundaries annihilate, is followed by a stable phase, in which the number of sectors does not change [4]. This behaviour in the 2D system is the consequence of initial merging of sectors, due to the diffusion of sector boundaries and the expansion of the radial frontier, which is inflating the distance between the sector boundaries. This results in the crossover time $t^{*}\sim R_{0}/v$ after which the coarsening is suppressed [54]. Here, $R_{0}$ is the radius of the initial “homeland”, which determines the length-scale of the initial sectors, and $v$ is the expansion speed of the radius. In a 3D colony growth, the sector boundary on the 2D surface is a fluctuating line, and a new domain can “pinch off” to form a new sector (e.g. [55]). When we ignore the effect of the roughness of the surface, the surface dynamics of the two-species Eden growth model are expected to show the same dynamics as the voter model [40, 54]. For the voter model on a flat surface, the coarsening dynamics over time, $t$, is marginal, where the density of the boundary between the sectors decreases in proportion to $1/\ln t$. This is because there is no “surface tension” to keep the sector boundaries compact. Even though merging of sectors occurs, the boundary of a large sector can fluctuate locally and segment into small sectors [56]. In the spherical expansion, the increase of the surface area is expected to suppress the already marginal coarsening. It should be noted, however, that the total surface area is increasing proportional to $t^{2}$. The number of sectors per area (i.e. surface density) in the Eden growth model showed a decreasing tendency over $t$ especially for the high-seed concentration (Supplementary Figure S10). This suggests that the expansion of existing sectors dominates most of the surface. In addition, it is worth noting that the roughness of the surface can affect the coarsening dynamics [57]. However, further theoretical studies are needed to characterize mixing and demixing dynamics in 3D range expansion quantitatively.

As our method is both cheap and easy to set up, this can be a natural extension of the original 2D competition assay to study 3D dynamics. The method can also provide a platform for a broad range of investigations with the potential for high throughput experiments [58]. Furthermore, this model system could be used to resolve not only how bacterial competition drives spatio-genetic patterning in neutral competition, but also how selective advantage, competitive interaction, cooperation and division-of-labour among the different cells distort this pattern in 3D growth by introducing different interacting cell types [14, 59–61]. Therefore, we anticipate that the proposed accessible method can help advance our understanding of microbial community development and evolution in related systems in 3D environments significantly.

## Supplementary Material

movie1_ycae009

movie2_ycae009

movie3_ycae009

movie4_ycae009

movie5_ycae009

movie6_ycae009

movie7_ycae009

movie8_ycae009

SI_ycae009

## Data Availability

All data and detailed protocols are available upon reasonable request and code is available on Zenodo (doi: 10.5281/ZENODO.8245914).
