# Supplementary figures and images for: Genetic mixing and demixing on expanding spherical frontiers
Source: ISME Commun. 2024 Jan 22;4(1):ycae009. doi: 10.1093/ismeco/ycae009 (PMC10958774; doi:10.1093/ismeco/ycae009)

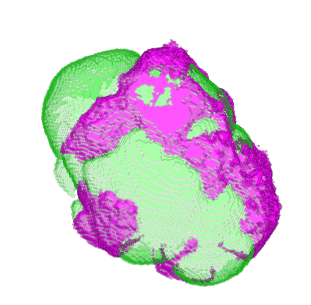

Supplement: movie1_ycae009 [file movie1_ycae009.gif]

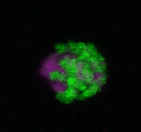

Supplement: movie2_ycae009 [file movie2_ycae009.gif]

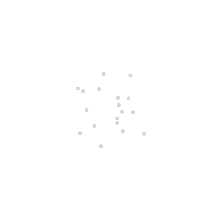

Supplement: movie3_ycae009 [file movie3_ycae009.gif]

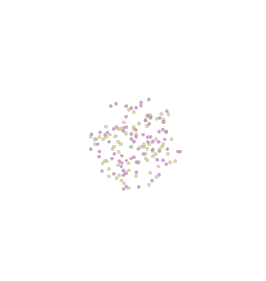

Supplement: movie4_ycae009 [file movie4_ycae009.gif]

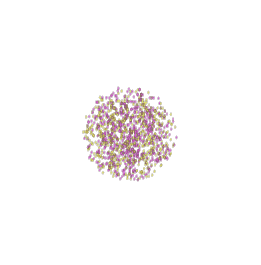

Supplement: movie5_ycae009 [file movie5_ycae009.gif]

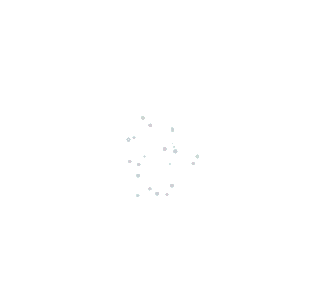

Supplement: movie6_ycae009 [file movie6_ycae009.gif]

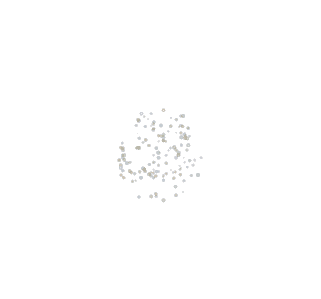

Supplement: movie7_ycae009 [file movie7_ycae009.gif]

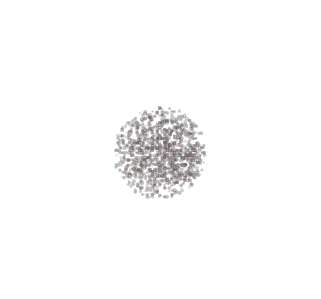

Supplement: movie8_ycae009 [file movie8_ycae009.gif]
